# Time tracking and comparison of genetic counseling tasks in inpatient and outpatient settings

**DOI:** 10.1002/jgc4.1935

**Published:** 2024-06-23

**Authors:** Alexandra Osborne, Emily Magness Bland, Callie Diamonstein, Kristen Fishler

**Affiliations:** ^1^ Munroe‐Meyer Institute for Genetics & Rehabilitation University of Nebraska Medical Center Omaha Nebraska USA; ^2^ Department of Molecular and Human Genetics Baylor College of Medicine Houston Texas USA; ^3^ Medical City Children’s Hospital Medical City Dallas Hospital Dallas Texas USA; ^4^ Present address: Oklahoma Children's Hospital University of Oklahoma Health Oklahoma City Oklahoma USA

**Keywords:** critical care, genetic counseling, multidisciplinary, professional development, service delivery models, time tracking

## Abstract

Genetic counselors (GCs) practice in critical care settings. Some GCs have full‐time inpatient roles, while most GCs who see inpatients do so as needed or on a rotating schedule in addition to seeing patients in an outpatient setting. Few studies have tracked and compared the amount of time it takes GCs to perform tasks in the inpatient and outpatient settings. Genetic counselors were invited to participate in this study via the National Society of Genetic Counselors research listserv. Participants completed an online survey asking how their role is structured and what types of support are available to them while seeing inpatients. They also performed time tracking for 16 tasks known to be associated with inpatient and/or outpatient care via RedCap. These tasks include direct patient care, care coordination, and other tasks which encapsulate a new patient encounter from beginning to end. Forty‐two inpatient encounters and 26 outpatient encounters were analyzed. The total average time spent on an inpatient consult (3 h and 38.5 min) was significantly higher than the time spent on an outpatient consult (2 h and 24.7 min; *p* < 0.05). Individually, genetic counselors spent significantly more time on the following tasks in an inpatient setting: direct follow‐up encounters, multidisciplinary team communication, results disclosure encounters, results disclosure documentation, traveling, and waiting. Follow‐up encounters, traveling, and waiting happen almost exclusively in inpatient settings. Short answer prompts regarding structure of GC role and available support revealed themes including lack of inpatient role structure, challenges with balancing between inpatient and outpatient tasks, and varied institutional support. These results promote further discussion about how to support GCs who see inpatients as these roles expand. Some suggestions include increased FTE/protected time and/or GCA support specific to the inpatient role.

**What is known about this topic**

Genetic counselors (GC) see patients in critical care settings. Many see inpatients as needed or on a rotating schedule, in addition to providing care to outpatients.

**What this paper adds to the topic**

The total average time spent on an inpatient consult was significantly higher than the time spent on an outpatient consult. Direct follow‐up encounters, multidisciplinary team communication, results disclosure encounters, results disclosure documentation, traveling, and waiting took significant more time in the inpatient setting compared to the outpatient setting. As the need for inpatient genetic counseling grows with increased access to genomic testing in critical care settings, protected FTE and/or GCA support may help GCs practice at the top of their scope.

## 1 INTRODUCTION

Recent studies have shown that interprofessional care in inpatient settings provides multiple benefits including positive patient health outcomes and increased satisfaction (Kleinpell et al., 2019). Previous research has shown that many patients admitted to a hospital have genetic etiology of their condition(s). Approximately one in three pediatric hospitalized patients have confirmed genetic disease or a genetic susceptibility to disease (McCandless et al., 2004). There are many studies which have identified the cost savings and clinical benefit of genomic sequencing for critically ill infants (Dimmock et al., 2020; Farnaes et al., 2018; Krantz et al., 2021; Petrikin et al., 2018; Stark et al., 2017). Awareness of the utility, payor coverage, and provider knowledge and experience ordering genomic testing has led to increased availability of this testing in the inpatient setting. Increased adoption of this testing requires increased genetics specialist support, including genetic counselors. Genetic counselors are distinctly qualified to contribute to critical care in inpatient settings with training in genetics and crisis counseling (Ayres et al., 2019; Lynch et al., 2021; Nisselle et al., 2019).

According to the 2023 National Society of Genetic Counselors (NSGC) Professional Status Survey (PSS), 53% of genetic counselors provided direct patient care and a portion of these genetic counselors practiced in inpatient settings (National Society of Genetic Counselors, 2023). The 2023 PSS asked genetic counselors to provide the percentage of their caseload spent seeing patients in an inpatient setting. There were 627 genetic counselors who provided any care to inpatients; most (474/627; 76%) of whom provided this care rarely (less than 25% of their caseload). Only 15 respondents provided care to inpatients as 100% of their caseload (National Society of Genetic Counselors, 2023). Previous research has demonstrated that genetic counselors see inpatients or cover an inpatient consultation service as needed or on a rotating schedule, often in addition to their primary full‐time obligations including outpatient care (Magness et al., 2021). A previous publication identified that on average, genetic counselors in outpatient settings providing pediatric or adult genetic counseling spent 2.3 h on patient‐related tasks per 1‐h patient slot (McPherson et al., 2008). A more recent study looking at genetic counseling in outpatient settings showed that GCs spent 3 h on patient‐related tasks per 47 min of face‐to‐face time with a patient (Attard et al., 2019). Research exploring genetic counseling service delivery across multiple specialties showed that genetic counselors need additional education, tools, and resources when implementing or modifying service delivery (Greenberg et al., 2020). However, there are no publications exploring the time spent and resource needs specific to inpatient genetic counseling.

This research study aimed to identify and compare the time spent on different tasks in the context of inpatient and outpatient encounters, and resource needs of inpatient genetic counselors. We hypothesized that overall a greater amount of time is spent on an inpatient encounter compared to an outpatient encounter. We also anticipated that some tasks would take different amounts of time in different settings. Examining the time spent on various tasks may help genetic counselors who see inpatients to advocate for protected time and additional resources to provide this care.

## 2 METHODS

### 2.1 Participants

Genetic counselors in North America were invited to participate in this study via the National Society of Genetic Counselors (NSGC) listserv. All genetic counselors who see inpatients in any specialty were eligible to participate, regardless of whether this role is officially incorporated into their Full‐Time Equivalent (FTE). Participants were also informed of the research listing via word of mouth at online inpatient special interest group (SIG) meetings, pediatric/clinical SIG meetings, and at the NSGC Annual Conference in the Fall of 2021. Participation in this research was voluntary. Responses were collected through REDCap from October 2021 to February 2022.

### 2.2 Measures and outcomes

We aimed to measure outcomes commonly seen in time tracking research: (1) average time spent on the total encounter across all genetic counselors in the study including when the participant recorded “0” as a time spent (total mean), which allows for direct comparison of time between the inpatient and outpatient encounters, (2) average number of minutes the task took to complete for those who performed the task in either setting (i.e. excluding participants who recorded “0” as a time spent for that task‐participation mean), and (3) the portion of the population who participated in the task (participation rate; Fisher et al., 2012). In this study, the term “time” is defined as the number of minutes needed to complete a task. Tasks include patient facing and non‐patient facing activities associated with providing genetic counseling services.

### 2.3 Instrumentation

A 20‐question survey was developed in REDCap (Research Electronic Data Capture) which is a secure, web‐based application designed to support data capture for research studies (Harris et al., 2009). Survey questions were adapted from a previous study characterizing the role of inpatient genetic counselors (Magness et al., 2021). Demographic variables included age, gender, race, years practicing as an inpatient genetic counselor, specialty, full‐time or part‐time status in their position, if inpatient work is something included in their contract, type of institution they work for, and region. Position‐specific variables included percentage of time spent in inpatient care, number of genetic counselors practicing in inpatient settings at their institution, and which specialists are present on the inpatient care team. We added an open‐ended question to explore the types of support genetic counselors receive to complete work‐related tasks, as this may impact time spent on administrative tasks. Participants were also asked to comment on their experiences with time management in their role.

Following the initial survey, participants were directed to a time tracking spreadsheet embedded within REDCap and were asked to record the time, in minutes, they spent on 16 tasks in inpatient and outpatient settings (if applicable) for their next five patient consultations in each setting. The definitions for these tasks were included on the time tracking page in REDCap and were designed to encapsulate an entire patient encounter from beginning to end (Appendix 1). The term “consultation” is defined in this study as an entire patient encounter from beginning to end and includes non‐patient facing tasks. Outpatient encounters recorded in this study could have included patients who were initially seen as inpatients who presented to outpatient care for follow‐up. For inpatients, the “end” point of the time tracking occurred at the pass‐off to the outpatient team following result disclosure and/or discussion of follow‐up care. For outpatients, the “end” point occurred after a follow‐up plan was created or after it was decided that no follow‐up is needed, after notes were signed, and no outstanding work remained for the outpatient visit. Participants who completed both the survey and time tracking spreadsheet were entered into a drawing for one of two $25 gift cards.

The research materials were piloted with a group of three inpatient genetic counselors. Revisions to the response options, wording of the questions, and task descriptions were made. Data gathered from piloting was not included in the analysis. Genetic counselors who participated in the pilot were also invited to participate in the study. This study was deemed exempt by the University of Nebraska Medical Center Institutional Review Board (IRB # 0566‐21‐EX).

### 2.4 Data analysis

Data collection occurred from October 2021 through February 2022. Participants were required to answer all survey questions to submit the demographic survey; no demographic data was excluded from analysis. Demographic data were reported as averages. Patient encounters dated before the study started and incomplete time tracking responses were excluded.

Time tracking data was analyzed in multiple ways, guided by previously published methods on performing time use studies (Gershuny et al., 2020). First, averages for the whole encounter and per task were calculated from all participant's time records, giving total mean. This included participants who did not complete the task (those documented times of 0 min), as it is possible that not all tasks were completed for each patient documented by the same genetic counselor. The Wilcoxon rank sum test was used to compare time between inpatient and outpatient tasks. A *p*‐value <0.05 was significant. Within‐participant comparisons were not performed due to small sample size. To calculate the total average time per task (in minutes), averages were calculated only from participants who performed the task (entered a non‐zero value), which provided the participation mean. The frequency at which participants performed each task in patient encounters was also calculated from participants who performed the task, which provided the participation rate. Responses to the two open‐ended questions were analyzed using descriptive thematic analysis and inductive coding approaches (Braun & Clarke, 2006).

## 3 RESULTS

A total of 25 genetic counselors who practiced within the United States or Canada responded to the survey. Of these, 11 performed time tracking. Among these genetic counselors, 42 inpatient encounters and 26 outpatient encounters were recorded.

### 3.1 Demographics

Participant demographics are shown in Table 1. Most participants were White (83%) and/or female (96%). The majority were 32 years of age or less (66%). Seventy percent of participants had been practicing in direct patient care as a GC for 5 years or less. Most participants had worked in the inpatient setting for four or fewer years (78%). Genetic counselors from all six United States geographic regions were represented in this data. About half of genetic counselors in this study (52%) practiced within an academic medical center. Many genetic counselors who provide inpatient service (36%) utilize a rotating schedule to cover the inpatient consultation service. Forty‐eight percent of respondents work with 1–3 other genetic counselors at their institution. However, 28% reported that they are the only genetic counselor providing inpatient services at their institution. Most genetic counselors (84%) share responsibilities with other providers and learners. The majority (88%) of genetic counselors have outpatient duties in addition to their inpatient role.

**TABLE 1:** Participant demographics.

Variable	*n*	% of responses
Age		
23–27	7	28
28–32	9	36
33–37	7	28
38–42	1	4
53–57	1	4
Gender^a^		
Female	24	96
Male	1	4
Race/Ethnicity^b^		
Asian	1	4
Hispanic, Latino, or of Spanish Origin	1	4
White	21	84
Other	2	8
Region		
Region 1: CT, MA, ME, NH, RI, VT, CN, Maritime Provinces	1	4
Region 2: DC, DE, MD, NJ, NY, PA, VA, WV, PR, VI, Quebec	6	24
Region 3: AL, FL, GA, KY, LA, MS, NC, SC, TN	4	16
Region 4: AR, IA, IL, IN, KS, MI, MN, MO, ND, NE, OH, OK, SD, WI, Ontario	6	24
Region 5: AZ, CO, MT, NM, TX, UT, WY, Alberta, Manitoba, Sask	3	12
Region 6: AK, CA, HI, ID, NV, OR, WA, British Columbia	5	20
Years in direct patient care as a GC		
<3	9	36
3–5	8	32
6–8	3	12
9–11	5	2
Time working in an inpatient setting (in years)		
<1	5	20
1–4	13	52
+5	7	28
Main specialty		
General Pediatrics	13	52
Neonatal	4	16
Pediatric Cardiology	1	4
Adult Cardiology	2	8
Metabolic	1	4
Cancer	3	12
Other		
Pulmonary	1	4
Additional areas of practice^c^		
General Pediatrics	18	72
Neonatal	10	40
Neurology	8	32
Pediatric Cardiology	11	44
Adult Cardiology	8	32
General Adult	12	48
Metabolic	7	28
Prenatal	2	8
Cancer	5	20
Pediatric Cancer	2	8
Other	4	16
Type of institution^c^		
Academic Medical Center	13	52
Public Hospital	7	28
Private Hospital	8	32
Utilization of GC services in inpatient care		
Dedicated GC for inpatient consults	7	28
Rotating schedule	9	36
Cover as needed	7	28
Other	2	8
Are your inpatient service responsibilities shared with others?		
Yes	21	84
No	4	16
Who are your inpatient responsibilities shared with?^c^		
Geneticists	20	80
GC assistant	2	8
Medical student	1	4
GC student	3	12
Fellow	9	36
Resident	9	36
Number of additional inpatient GCs at institution		
0	7	28
1–3	12	48
4–7	6	24
Do you also have outpatient duties?		
Yes	22	88
No	3	12

^a^ Other answer options which were not chosen by any participant: transgender female, transgender male, non‐conforming, prefer not to answer, and other.

^b^ Other answer options which were not chosen by any participant: American Indian or Alaska Native, Black or African American, Native Hawaiian or Other Pacific Islander. Participants could only choose one option. For this question, the stem asked participants, “How would you describe yourself?” We acknowledge the answer options included examples of races and ethnicities. The authors have made changes to their processes for collecting demographic information to be more inclusive and accurate going forward.

^c^ Respondents could select all that apply, % calculated from *n* over total number of respondents (*N* = 25).

### 3.2 Time tracking

**FIGURE 1:**
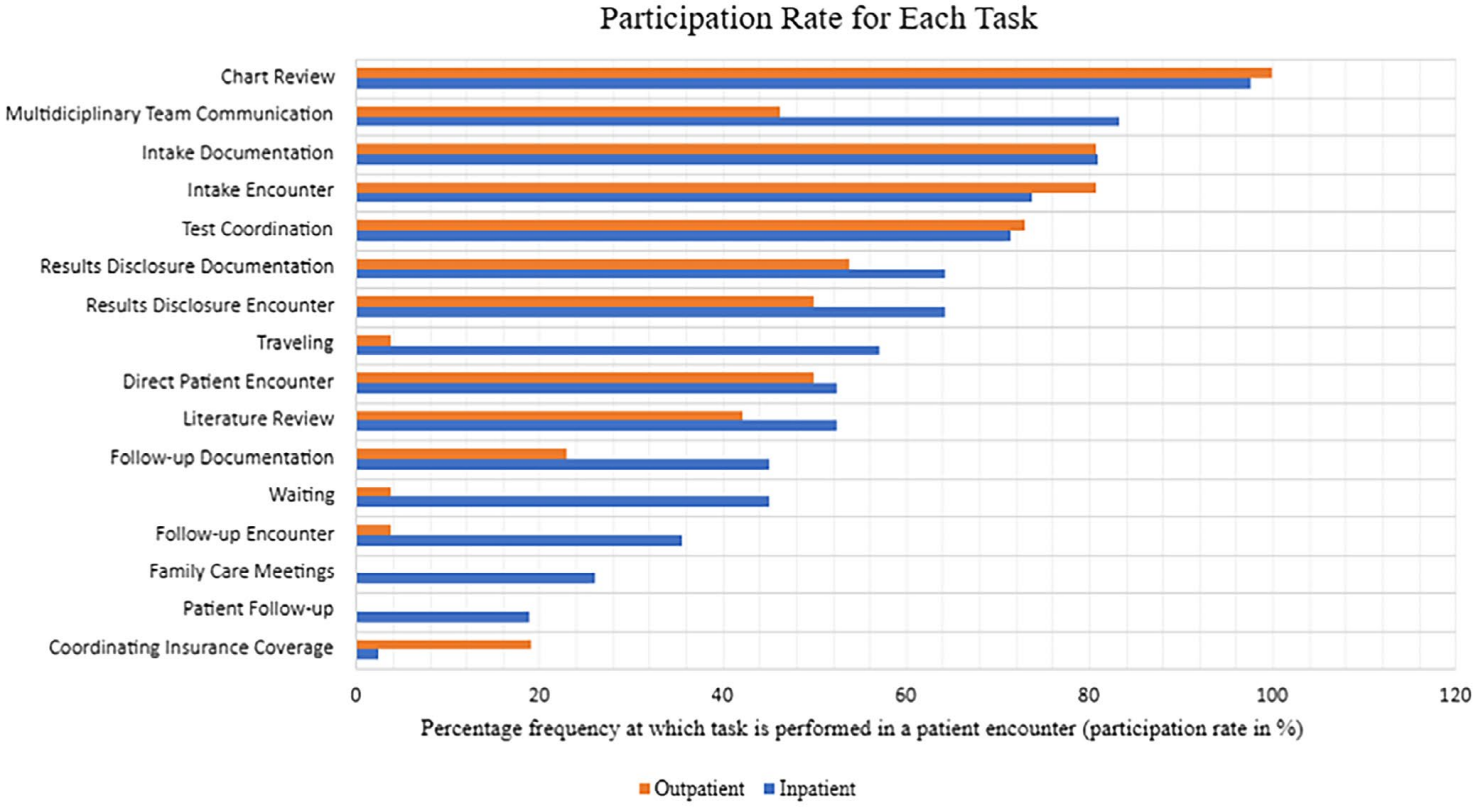
Participation rate. This bar graph indicates the percentage of participants who performed the task in a patient encounter in each setting.

Time tracking comparisons across all participants are reported in Table 2 (total mean). Overall, total time spent on an inpatient genetic counseling encounter is higher than an outpatient encounter (*p* < 0.05). The average inpatient consultation took 3 h and 38.5 min while the average outpatient consultation lasted 2 h and 24.7 min. Inpatient consultations ranged from 49 to 580 min (9 h, 40 min). Outpatient consultations ranged from 70 min (1 h, 10 min) to 430 min (7 h, 10 min). Average time spent per task for participants who performed each task is summarized in Table 3 (participation mean), which provides the average number of minutes the task would take. Participation rates of genetic counselors who performed each task are summarized in Figure 1. This represents the proportion of the study population completing each task during a patient encounter. Direct follow‐up encounters, family care meetings, patient follow‐up, traveling, and waiting were tasks completed almost exclusively in the inpatient setting.

**TABLE 2:** Total mean – comparison of average time spent between genetic counseling tasks in the inpatient and outpatient settings.

	Inpatient (minutes)	Outpatient (minutes)	*p*‐Value
Total encounter time	218.5 (140.7)	144.9 (75.6)	**0.016**
Chart review	24.6 (20.2)	30.0 (35.3)	0.641
Intake encounter	19.5 (16.6)	27.4 (18.9)	0.073
Intake documentation	17.9 (17.6)	14.1 (11.9)	0.508
Direct patient encounter	21.6 (32.6)	19.0 (23.1)	0.995
Direct follow‐up encounter	6.1 (10.5)	0.2 (1.0)	**0.024**
Follow‐up documentation	6.9 (9.2)	7.9 (16.1)	0.350
Multidisciplinary team communication	15.5 (16.0)	5.3 (7.1)	**0.001**
Coordinating insurance coverage	0.8 (4.7)	2.2 (6.3)	0.325
Test coordination	16.7 (22.3)	10.6 (10.3)	0.405
Literature review	16.4 (20.9)	13.5 (25.9)	0.370
Results disclosure encounter	19.9 (23.4)	6.9 (8.5)	**0.020**
Results disclosure documentation	16.1 (18.7)	6.9 (9.0)	**0.048**
Traveling	8.8 (9.4)	0.2 (1.0)	**<0.001**
Waiting	11.7 (18.2)	0.8 (3.9)	**0.004**

*Note*: Averages were calculated from all participant time records including those who reported 0 min spent on the task to account for variability across the population. Thus, the time listed per task represents the total mean across the population, as not all tasks are completed every encounter. Standard deviations are reported in parentheses. Comparisons were analyzed by the Wilcoxon rank sum test. Tasks only performed in the inpatient setting (family care meetings and patient follow‐up) were removed from this table. Bolded numbers in the p‐value column are for tasks where the *p*‐values were <0.05, which is our level of significance.

**TABLE 3:** Participation mean‐average time spent per task, in minutes, in each setting.

	Inpatient, *N*	Minutes in the inpatient setting (SD)	Outpatient, *N*	Minutes in the outpatient setting (SD)	*p*‐Value
Total encounter time	42	218.5 (140.7)	26	144.9 (75.6)	**0.016**
Chart review	41	25.2 (20.1)	26	30.0 (35.3)	0.757
Intake encounter	31	26.5 (13.6)	21	33.9 (14.7)	0.052
Intake documentation	34	22.1 (17.1)	21	17.5 (10.7)	0.372
Direct patient encounter	22	41.2 (35.1)	13	38.1 (18.0)	0.670
Direct follow‐up encounter	15	17.2 (11.0)	1	5.0 (−)	0.278
Follow‐up documentation	19	15.3 (7.7)	6	34.2 (15.0)	**0.003**
Multidisciplinary team communication	35	18.6 (15.8)	12	11.5 (6.0)	0.172
Family care meeting	11	43.0 (24.4)	0	‐ (−)	
Coordinating insurance coverage	2	17.5 (17.7)	5	11.2 (11.2)	0.699
Test coordination	30	23.4 (23.3)	19	14.5 (9.3)	0.124
Literature review	22	31.2 (19.2)	11	31.8 (32.2)	0.541
Results disclosure encounter	27	30.9 (22.6)	13	13.8 (6.9)	**0.002**
Results disclosure documentation	27	25.1 (17.9)	14	12.8 (8.5)	**0.006**
Patient follow‐up	8	24.4 (19.4)	0	‐ (−)	
Traveling	24	15.4 (7.2)	1	5.0 (−)	0.145
Waiting	19	25.9 (19.2)	1	20.0 (−)	1.000

*Note*: Participation mean. The average time (in minutes) of those who performed the task in each setting (i.e. including only those participants who spent >0 min on the task). Comparisons were analyzed by the Wilcoxon rank‐sum test. Standard deviations are reported in parentheses.

Bolded numbers in the p‐value column are for tasks where the p‐values were <0.05, which is our level of significance.

### 3.3 Open‐ended responses

Participants were asked to comment on their experiences with time management and the types of support they receive to perform duties related to their inpatient role. Thematic analysis revealed three major themes: (1) lack of inpatient role structure/organization, (2) challenges with role switching between inpatient and outpatient tasks, and (3) varying levels of institutional support.

#### 3.3.1 Theme 1: Lack of inpatient role structure/organization

Many respondents discussed the limited structure, organization, and awareness of inpatient genetic counseling roles within their institution. For example, one participant commented that their “inpatient teams are not fully aware of the role of a genetic counselor which can be frustrating and lead to miscommunications.” Another respondent mentioned that “inpatient work is not well scoped or scheduled… we never know whether an inpatient will need GC services, geneticist, resident, or other specialty.”

Additionally, participants mentioned that disorganization and lack of structured workflow led to inefficiencies in their role. For example, one participant stated: “When we are not fully staffed, we rotate ‘on call’ days throughout the week and will frequently see outpatients on ‘on call’ days. This is much harder to manage and can lead to inpatient needs not being met. I love inpatient genetic counseling and wish it was a more structured role at more hospitals.” Another participant commented that the inpatient role at their institution “… is not very organized and can be extremely variable. It is also harder to follow up on results/follow up items because of the lack of organization.”

#### 3.3.2 Theme 2: Challenges with balancing inpatient and outpatient tasks

Several participants brought up challenges with time management in the inpatient setting. Specifically, they mentioned difficulty in juggling tasks across inpatient and outpatient roles within the same day. One participant stated: “There are many roadblocks and competing factors in management including finding time to talk to a family, correspond with other consulting specialties, and working with geneticists with competing schedules to see patients.” Other respondents with combined inpatient and outpatient roles mentioned the difficult balancing act of “shift[ing] your schedule and making [inpatient consults] a top priority because that testing is usually more time sensitive” and “being given inpatient consults and expected to go immediately in the middle of clinic to the floor.”

Participants also mentioned that follow‐up tasks for inpatients sometimes continued outside of scheduled or protected time for inpatient duties. For example, one individual stated: “even on days I am not on‐call, I am following up on patient results, coordination, and psychosocial check‐ins.” Additionally, a concern was raised about balancing inpatient roles with education roles, “…I do not feel our institution has a good understanding of the time required to appropriately manage inpatient consults and in managing all the learners.”

#### 3.3.3 Theme 3: Varying levels of institutional support

Participants commented on varying levels of support within their institution. A few stated that they receive little support from their institution to perform their inpatient tasks. For example, one participant commented “…there is little to no support in GC's covering inpatient services. It is looked at as part of our job (which it is), but there is little acknowledgement of our current workload for that day.” This statement may also be highlighting concerns about inpatient roles not being included in their Full‐Time Equivalent (FTE). As one participant stated: “we do not have any FTE to support this role. It is a part of our expected responsibilities.”

Others mentioned that support is dependent on team structure and leadership. Two participants mentioned that other staff members and learners on the inpatient team are useful sources of support. They stated: “Our inpatient medical director supports us using our time in whatever way we need to … When we have a medical student, fellow, and/or resident, some tasks are delegated to them to help with their training” and “My colleagues are very supportive and are willing to help if a day gets overwhelming.” Although, as previously mentioned, sometimes working on a team can lead to miscommunication due to lack of understanding of the role of a genetic counselor in the inpatient setting.

To assist with offloading time for genetic counselors, three participants mentioned the role of Genetic Counseling Assistants (GCAs) to moderate workload in the inpatient setting. One participant stated, “We have a GCA and medical assistant that can help request records as needed.” Another genetic counselor mentioned they utilize GCAs for coordination of parental follow‐up testing in the inpatient setting.

## 4 DISCUSSION

This study aimed to characterize the time it takes a genetic counselor to perform genetic counseling tasks in an inpatient setting and compare the time it takes to perform these tasks in an outpatient setting. This study also aimed to learn more about the structure of inpatient genetic counseling roles and the types of support they receive through open‐ended responses. Our results indicate that more time is spent completing various genetic counseling tasks in the inpatient setting compared to the outpatient setting. Tasks that took significantly more time in the inpatient setting included: direct follow‐up encounters, multidisciplinary team communication, results disclosure encounters, results disclosure documentation, traveling, and waiting. The open‐ended responses identified that genetic counselors in inpatient settings work with a variety of providers and learners with different team structures. Results from this study show that most participants cover consults on an “as needed” basis, or on a rotating schedule, which is consistent with previous literature (Magness et al., 2021).

Traveling and waiting were tasks that many genetic counselors spent time on in the inpatient setting. These tasks include walking to and from the inpatient unit and waiting for other providers to exit the patient room. These tasks were not documented for any outpatient encounter. Ways to reduce the amount of time genetic counselors are “waiting” to provide care may include contacting the bedside nurse and/or managing provider to coordinate visits with the family or at bedside. Additionally, integrating telehealth with audio or audio and video for inpatients, even in locations the GC can physically visit, may be another feasible way to reduce GC waiting time. A telehealth appointment is something the family can decide regarding timing and who else they would like to attend the visit, including other family members who may not be allowed at the bedside due to contact or other restrictions. In addition, it may help facilitate time efficiency for the genetic counselor as they can add multiple telehealth visits to their schedule on particular days. By “blocking” time for telehealth visits, this may also help better define how a genetic counselor in the inpatient setting is spending their time for tracking and potential billing purposes. Previous research has demonstrated the utility of telehealth for providing care to patients in geographically isolated areas, in isolation for viral illness, or in emergency situations where it is not possible for the provider to physically make it to the bedside (Burke & Hall, 2015; Umoren et al., 2020). A recent study in Nebraska also identified high patient satisfaction with telehealth, with most visits including audio and video, and a desire to receive future care by telehealth, even for patients who live within the catchment area of a genetics clinic (Rezich et al., 2021). There are no studies, however, which investigate the acceptability, feasibility, or time saving of offering telehealth to families for patients in an inpatient setting.

Only 26% of participating genetic counselors in this study attended family care meetings. When this was performed, however, an average of 43 min was spent on this task. Family care meetings are inherent to the role of a genetic counselor in the inpatient setting and are known to dramatically impact care for parents with an acutely ill infant (Aldridge et al., 2021; Ayres et al., 2019; Haward et al., 2020). Of note, this task was only reported in the inpatient setting so direct comparisons between inpatient and outpatient settings were not possible. It is not known why most genetic counselors in this study did not participate in this task, although it should be noted that these meetings may not occur for all patients. Thus, it is possible that genetic counselors who did not complete this task during the time tracking may attend family care meetings when organized, but that it was not part of patient care for the cases they performed time tracking for, which may lead to under‐reporting of participation in this task. The COVID‐19 pandemic was ongoing during this study, which may have further limited opportunities for family care meetings or other tasks in this setting. Future research inquiring about inpatient genetic counseling scope and practice should inquire further about whether most genetic counselors participate or are asked to attend family care meetings and how this impacts their time seeing patients in this setting.

Genetic counselor's responses regarding institutional support for their inpatient role varied. Some stated they receive little support while others commented that support is dependent on team structure and may be moderated by other provider's knowledge and awareness of the role of a genetic counselor in the inpatient setting. Thematic analysis shows that the role of a genetic counselor as part of an inpatient care team may not be universally understood, which can lead to challenges for genetic counselors in this setting. This may point to more opportunities for education of non‐genetics inpatient providers as a genetic counselor in this setting. It may also point to a need for more administrative support for genetic counselors who see inpatients.

Some genetic counselors commented on the use of support staff including GCAs. Genetic counseling assistants (GCA) have been utilized in other areas of genetic counseling practice to reduce GC burden associated with tasks including test coordination and insurance coverage (Pirzadeh‐Miller et al., 2017). Including GCAs on a cancer genetic counseling team has been associated with increased patient volume and decreased time spent in appointments (Hallquist et al., 2020). Additional studies have identified that a GCA's involvement including things such as assisting patients with a pre‐session clinical survey, add standardized templates to notes, collecting specimens (and calling to remind them to collect them), entering appointment information into a database, and performing pre‐authorization, did not have a negative impact on patient care in this setting, (Cohen et al., 2023). Implementation of a GCA in the inpatient setting may help offset time GCs spend taking care of patients in this setting to allow them to see more patients. To date, the role of a GCA has not been explored in the inpatient setting. Anecdotally, GCAs in this setting may perform result follow‐up, parental sample coordination for genomic testing, patient scheduling, patient database tracking, tracking patient follow‐up in an outpatient setting, and other tasks. Further exploration into the role of an inpatient GCA is necessary, specifically as it pertains to GC time spent in this role per patient and patient satisfaction as well as how integrating a GCA may impact psychological variables for the genetic counselor, such as burnout and compassion fatigue.

When asked about the structure of their inpatient role, many genetic counselors commented on their challenges with managing time between their inpatient and outpatient roles. Genetic counselors commented that they often shift their schedule or drop what they are doing in outpatient clinics to tend to the fast‐paced critical nature of inpatient care. They also mentioned spillover of inpatient tasks to days when they were not “on‐call” or had protected time to see inpatients. This could result in delay of outpatient care, provider anxiety, or reduction of quality of care to accommodate inpatient volume. Like many other health care providers, genetic counselors are at risk for high levels of stress and burnout (Caleshu et al., 2022; Lee et al., 2015). In critical environments, such as the intensive care units, proper structure and organization must be in place to alleviate compounding stressors for providers. Patient satisfaction has been shown to positively corelate with hospital staff job satisfaction (Linn et al., 1985; Nurmeksela et al., 2021). Additionally, interventions focused on process improvement have been noted to reduce job‐related stress and decrease burnout in healthcare settings (DeChant et al., 2019). While adverse work conditions negatively impact patient care, add to occupational stress, and lead to burnout among healthcare workers (Angerer & Weigl, 2015; Chandawarkar & Chaparro, 2021; Linzer et al., 2009). Decreasing cost of care is another positive outcome associated with better management of inefficiency and variability in care (Shrank et al., 2019). Altogether, these findings advocate for process improvement for inpatient genetic counselors including additional structure, organization, and definition of roles.

One solution to preventing adverse effects, due to switching between inpatient and outpatient roles while also potentially enhancing patient care, includes provision of protected time or increase in the full‐time equivalent for genetic counselors providing care to inpatients. This may help genetic counselors balance the tasks between inpatient and outpatient roles and provide comprehensive and appropriate care in both settings. Some genetic counselors in this study mentioned that they do not have protected time for care provided to inpatients. One way for administration to define time spent and moderate workload is through Full‐Time Equivalent (FTE). However, it is well known that accurately measuring FTE can be challenging for many healthcare providers in inpatient and outpatient settings (Gallagher & Rapoza, 2010; MacDonald et al., 2013). The National Society of Genetic Counselors Professional Status Survey is conducted yearly asking genetic counselors to define aspects of their role(s) and quantify how they spend their time, among other topics. One of the metrics includes the number of patients seen, which is reported by specialty (National Society of Genetic Counselors, 2023). Although the 2023 PSS asked genetic counselors to provide the percentage of time they spend seeing inpatients, it did not ask them to quantify how many inpatients they saw per week or per month. Further, interpretation of patient volumes from the raw PSS data would be challenging, given that most genetic counselors are not seeing inpatients for most of their direct patient care FTE. Even with these limitations, this data could be considered by clinical administration to quantify how many patients a genetic counselor should see to fulfill an inpatient FTE, given that the encounter time and specific tasks critical to the inpatient role such as follow‐up documentation and result disclosure encounters/documentation take more time in the inpatient setting. It may be inappropriate to hold genetic counselors seeing inpatients to the same patient volume as an outpatient genetic counselor, without considering the amount of time it takes to perform each task in each setting. Based on the average time it takes to perform one inpatient consultation (~3.5 h) in this study, seeing 11 inpatients per week would take up a 40‐h work week. This does not, however, consider any other responsibilities that may be part of a GC's workday such as answering phone calls or emails from patients or physician offices, reviewing referrals, etc. Therefore, it may be that the full‐time equivalent for an inpatient genetic counselor should be less than 11 new patients seen per week. Additional studies to assess patient load and FTE across institutions are warranted.

### 4.1 Study limitations

This data represents a small portion of all genetic counselors who see inpatients. While participants represented multiple institutions across the country in different regions, these aspects limit the generalizability of these findings. Not every participant tracked the maximum number of patient encounters as part of this study. Additionally, given the ongoing nature of inpatient encounters, several tasks may not be accounted for during the time tracking period of this study and are thus underrepresented. The COVID‐19 pandemic was ongoing during the time of this study, which may have limited opportunities for GCs to participate in research given additional demands for genetic counselors to fulfill different roles than they did prior to the pandemic as well as employment changes (MacFarlane et al., 2021; Wagner et al., 2021). The clinical constraints regarding the number of people who could gather with masks during the COVID‐19 pandemic may have also limited opportunities for family care meetings or other interactions in this setting during the time of this study, potentially leading to under‐reporting of time taken for tasks in inpatient and outpatient settings. Finally, interest in this study may have been limited by time, given the pandemic and that the 2023 PSS identified that most inpatient genetic counselors provide care to inpatient and outpatient populations simultaneously. Lastly, self‐report is a known limitation of time tracking and genetic counselors may have been motivated to over report on certain tasks, especially if they did not have protected time to provide inpatient services.

### 4.2 Practice implications

More time is needed for genetic counselors to provide inpatient care than performing the same tasks in an outpatient setting. This study identified the need for further characterization of FTE for GCs with inpatient roles and protected time for genetic counselors who also have outpatient or other roles. The addition of a GCA to the inpatient team may also help streamline workflow, reduce time spent on repetitive or below scope tasks, and reinforce support. Additional improvements could be education for bedside providers on the role of inpatient genetic counselors and clarification within the genetic team regarding geneticist vs. genetic counseling roles.

### 4.3 Future directions

This study shows that inpatient genetic counseling takes more time, however, it was not designed to analyze why this occurs. Given the low number of genetic counselors who participated in this study, a more robust time tracking study specific to inpatient genetic counselors and stratified by specialty is needed. Future exploration through qualitative interviews would allow for greater understanding of how genetic counselors are spending their time as well as barriers they may face in this setting. Future studies should include comparison of workflows that may moderate time spent to determine best practices in maintaining efficiency and a high level of patient care. Additional studies should be performed specific to inpatient genetic counselors regarding sources and moderators of burnout. This may include situations involving moral distress, patient volume, administrative support, and other factors. Further exploration of the role and integration of a GCA in the inpatient setting is also needed as it may help offset GC time.

## 5 CONCLUSION

This is the first study to assess the time it takes to perform genetic counseling tasks in an inpatient setting. It is also the first study to compare the time spent on these tasks with time spent on these same tasks in an outpatient setting. We demonstrated that the average time spent on tasks in the inpatient setting is higher than the time spent on tasks in an outpatient setting (*p* < 0.05). Furthermore, we discovered that genetic counselors found varied levels of support, disorganization, and understanding of their roles to be barriers in the inpatient setting. The results of this study argue for protected full‐time equivalents for genetic counselors providing inpatient care, consideration of how GCAs can support GCs in inpatient roles, and greater organizational structure for genetic counselors to facilitate role balancing for genetic counselors with inpatient and outpatient roles.

## AUTHOR CONTRIBUTIONS

Authors Alexandra Osborne and Kristen Fishler had full access to all the data in the study and take responsibility for the integrity of the data and the accuracy of the data analysis. All the authors gave final approval of this version to be published and agree to be accountable for all aspects of the work in ensuring that questions related to the accuracy or integrity of any part of the work are appropriately investigated and resolved.

## CONFLICT OF INTEREST STATEMENT

Alexandra Osborne, Emily Magness Bland, Callie Diamonstein, and Kristen Fishler declare that they have no conflict of interest.

## ETHICS STATEMENTS

Human studies and informed consent: This study was reviewed and granted an exemption by the University of Nebraska Medical Center IRB. All procedures followed were in accordance with the ethical standards of the responsible committee on human experimentation (institutional and national) and with the Helsinki Declaration of 1975, as revised in 2000. Implied informed consent was obtained for individuals who voluntarily completed the online survey and submitted their responses.

Animal studies: No non‐human animal studies were carried out by the authors for this article.

## Data Availability

The data supporting this study's findings are available from the corresponding author upon reasonable request.

## APPENDIX 1

### Definitions for time tracking spreadsheet

*Chart review*: looking through patient charts in preparation to see this patient.

*Intake encounter*: time spent with the patient during initial visit.

*Intake documentation*: paperwork that you are required or elect to fill out during an initial visit.

*Direct patient encounter*: time spent with patient or family not including intake or results disclosure encounters.

*Direct patient follow‐up encounter*: time spent with patient or family not including intake or results disclosure encounters following the initial direct patient encounter.

*Follow‐up documentation*: paperwork that you are required or elect to fill out following a patient encounter.

*Multidisciplinary team communication*: communication with any team member involved in patient care. This can be face‐to‐face, telephone, or email correspondence.

*Family care meetings*: meetings together with the patient, family, and health care team.

*Coordinating insurance coverage*: face‐to‐face, telephone, or email correspondence with individuals other than the patient/patient's family to coordinate insurance aspects.

*Test coordination*: time spent filling out forms or communicating about ordering genetic tests.

*Literature review*: seeking additional information on a condition, variant, practice guideline, or similar topic by searching current documented information or scholarly literature.

*Results disclosure encounter*: time spent disclosing results with patient and family.

*Results disclosure documentation*: time spent documenting results and the disclosure of these results to patients. Please place time spent actually disclosing results within the results disclosure encounter cell.

*Patient follow‐up*: any communication following patient discharge.

*Travel Time*: time spent traveling to and from patient appointments (this includes driving and/or walking from your office or prep area to the patient).

*Wait Time*: time spent waiting to see the patient (this includes things such as waiting for family to return to bedside and waiting for other providers to finish meeting with the patient).
